# What Is Homœopathy?*The writer of this article, after reading Dr. Wells on the International Homœopathic Convention, hesitated about having it put in print, but as truth never loses by having the light thrown upon it from different directions, he has concluded to publish it, even at the risk of having the Doctor suspect something may have been borrowed from him.

**Published:** 1881-12

**Authors:** C. H. Lawton

**Affiliations:** Wilmington, Del.


### WHAT IS HOMŒOPATHY?*

C. H. Lawton, M. D., Wilmington, Del.

This subject has been so often and ably discussed, that I cannot hope to add any thing of great value to our current literature, but there are always those who need to be encouraged and strengthened; there are those, who, unless the truth is kept constantly before them lose sight of it altogether.

When I read the reports of medical conventions and local societies, which represent our school, I am often led to exclaim: When will homœopaths teach and practice homœopathy? And the conviction is forced upon me, that to-day even among homœopaths, few have comprehended the beauty and grandeur of the sublime truths of homœopathic law.

To those who have no faith in homœopathy, as a vital principle, I have nothing to say.

The time for ridicule is past. Small doses and sugar pellets no longer constitute the essential part of homœopathy.

*Similia similibus curantur*, is no longer read like a guide-board, and immediately forgotten, and homœopaths are rapidly learning that this is the enunciation of a great truth, which faithfully followed is placing, and will place homœopathy far in the van of all curative law known among men.

Following with steady faith this bright star of promise, we shall emerge from darkness into light, from the thraldom of allopathy and eclecticism into the liberty of those whom the truth has made free.

There are men who have as a natural endowment, liberty of thought and action; progressive minds, which are always in advance of their fellows, ever seeking the highest and best; ever basking in the very sunlight of truth.

---

*The writer of this article, after reading Dr. Wells on the International Homœopathic Convention, hesitated about having it put in print, but as truth never loses by having the light thrown upon it from different directions, he has concluded to publish it, even at the risk of having the Doctor suspect something may have been borrowed from him.

Though always ready to accord the broadest liberty to others, they never demand it for themselves, for "whom the truth has made free are free indeed." Undaunted by ridicule; made strong by opposition; with an unfaltering faith in the right, no power on earth can check the progress of their thought.

Such a man was Hahnemann, such men were Hering and Dunham, such men are living to-day, and *while such men live* homœopathy can never die.

Love is the law of knowledge, and to form a proper conception of homœopathy we must love it, appropriate it, engraft it into our lives, let it grow with our growth, and strengthen with our strength; then having absorbed a great truth, we will feel no attraction toward error, can make no compromise with it, or for a moment parley with that which we know to be questionable or wrong. Not long ago a distinguished cotemporary in an address to a body of homœopathic physicians, said: "Homœopathy is a method; and it is the method of Hahnemann."

In answering the question—What is Homœopathy? I am compelled to take exception to this definition; it is robbing homœopathy of its birth-right; no man however great; no intellect however exalted and powerful; no genius however brilliant; no finite being ever originated a perfect law.

We may love, and honor, and reverence the great man who was the founder of our school, but a greater than Hahnemann is here.

Homœopathy is not an invention, but a discovery, a fundamental law, that has existed throughout all time. Hahnemann was its discoverer, but its author was the Infinite One. The methods of man are imperfect and open to criticism, but the laws of God are immutable and eternal.

Great men have lived and died, leaving to posterity legacies rich in thought and scientific research, but it remained for Hahnemann first to discover and comprehend this great truth himself, then to demonstrate it to others, with wonderful powers of perception and discrimination, systematizing and adapting it to the needs of suffering humanity. This *latter* is pre-eminently the *method* of Hahnemann.

Just here let me impress this fact: If our therapeutic law *Similia similibus curantur*, is a fundamental principle, and that it is no intelligent homœopathic physician at the present day doubts, it is true at all times and under all circumstances. *Let us prove homœopathy.*

If we would utilize hydropathy, let us use it homœopathically; if we prescribe changes in diet, let us do so on homœopathic principles; if our patient cannot sleep, let us remove the pathological condition which causes sleeplessness, by selecting and prescribing the appropriate homœopathic remedy; nor dare to put our judgment in opposition to an immutable law.

"*In that, we presume to see what is meet and convenient better than God himself.*"

Pardon me, if in this connection, I allude to a few facts from personal experience, for the encouragement of any who may desire to practice pure homœopathy, yet hardly dare venture on what may seem to them a doubtful experiment.

During the past ten years the writer has depended exclusively on our law of cure, never having *once* prescribed an alcoholic stimulant, or given a dose of purgative medicine, or used an anodyne to relieve pain or produce sleep; nor does he let his patients suffer, but has always been able to relieve them homœopathically. The average mortality being 2.7 per cent.

This brings the question of posology squarely before us, and although the writer is not an extremist, and has never been known as a high potency man, he must insist that no consistent homœopath can afford to presistently oppose, or ridicule, or ignore, this question.

To be ignorant, or prejudiced, or bigoted, while truth stands knocking at the door, and human lives are being weighed in the balance, is criminal.

It has been demonstrated that in both acute and chronic diseases the high potencies act more promptly than the low attenuations or tinctures.

These facts have been given to the world and need no urging in this paper. They are the conservators of truth and *will not* be ignored.

What the profession demands to-day is, a scientific solution of this problem.

In attempting to give this, I submit the following propositions: 1st. The primary effect of a drug prescribed homœopathically, acting as it does in the direction of the disease, must be a temporary aggravation. 2d. It is the secondary effect or reaction that cures. 3d. The shorter the duration of the primary effect, or aggravation, the more quickly do we get the secondary, or curative effect.

The more we simplify our exposition of a subject, the more readily will it be comprehended by others. That one and one are two is a very simple problem, but it is at the foundation of mathematical science.

That the primary effect of a small dose of medicine is of shorter duration than that of a larger, and that the vital forces react against it more promptly, is a very simple proposition, but it is an essential principle of our therapeutic law.

Let us see how this works practically. A young physician is called to prescribe for a patient suffering from an acute disease; being a good homœopath he prescribes according to the law of Similia, but in a low attenuation, or perhaps a few drops of the tincture in water.

The active principle of the drug never having been developed it acts slowly, and the patient is not relieved.

Under the erroneous impression that the low attenuations may be frequently repeated, he gives another dose with no better success.

What is to be done? His sympathies are aroused, his reputation is at stake; his patient is getting impatient; he must have *immediate relief*, and in the desperation of the moment the doctor gives a dose of morphia, or perhaps a hypodermic injection of the same drug. His patient is relieved.

Henceforth, he goes armed with a hypodermic syringe, and a supply of morphia.

But what has he *really* done? He has relieved his patient! but at the expense of his vitality; he has, by failing to comprehend and apply the best method embraced within our law of cure, exposed homœopathy to the sneer of opponents, and wounded it in the house of its friends, and worse than all he has lost faith in the efficacy of homœopathy, where prompt action is required, and has degenerated into eclecticism.

At the next sectional meeting he talks about "Freedom of Medical Opinion and Action."

It is a libel on the noble words of Carroll Dunham.

Suppose a potentized remedy had been given? The active principle of the drug having been previously developed by trituration, or succussion, there would have been a sudden impulse in the direction of the disease, which would have been quickly removed, followed by reaction and prompt relief.

Allopathy meets us as an open foe and can do no harm, but eclecticism among homœopaths is a delusion and a snare; it comes to us in the garb of a friend, and makes its demands in the name of liberty and philanthropy, yet offers in return nothing but empiricism. The delusion that "*it is our supreme duty to do what we judge best for our patients,*" *to select according to our judgment the best from all*

*schools* has seduced many a homœopath into the ranks of the eclectics.

In conclusion let us for a moment consider, and try to analyze the exalted sentiment of Carroll Dunham, quoted so frequently of late, and used to justify all kinds of promiscuous and unscientific practice. "*Freedom of Medical Opinion and Action.*"

Freedom of opinion must be based upon enlightened, unprejudiced, scientific thought, and must be in harmony with that which we know to be true. And knowing the truth, if we have the courage to proclaim and practice it, then have we "Freedom of Opinion and Action." If we know not the truth, or knowing, have not the courage to practice it, then are we slaves and not freemen.

To those who so urgently demand freedom of opinion and action, let me say no man can give, no man can take it from you.

The power that enslaves, sits regent in your own brain.

May we all strive after this liberty of thought, not by making demands where no equivalent can be given, but let us invite this child of reason to be our guest, to sit at our board, to *sup* with us, and let the feast be, *Union, Liberty, Charity and Love.*

